# Shared genetic risk factors and causal association between psoriasis and coronary artery disease

**DOI:** 10.1038/s41467-022-34323-4

**Published:** 2022-11-02

**Authors:** Matthew T. Patrick, Qinmengge Li, Rachael Wasikowski, Nehal Mehta, Johann E. Gudjonsson, James T. Elder, Xiang Zhou, Lam C. Tsoi

**Affiliations:** 1 Department of Dermatology, Michigan Medicine, University of Michigan, Michigan, MI USA; 2 Department of Biostatistics, School of Public Health, University of Michigan, Michigan, MI USA; 3 Section of Inflammation and Cardiometabolic Disease, National Heart, Lung, and Blood Institute, National Institutes of Health, Michigan, MD USA; 4 Department of Computational Medicine and Bioinformatics, Michigan Medicine, University of Michigan, Michigan, MI USA

**Keywords:** Risk factors, Skin diseases, Genome-wide association studies

## Abstract

Psoriasis and coronary artery disease (CAD) are related comorbidities that are well established, but whether a genetic basis underlies this is not well studied. We apply trans-disease meta-analysis to 11,024 psoriasis and 60,801 CAD cases, along with their associated controls, identifying one opposing and three shared genetic loci, which are confirmed through colocalization analysis. Combining results from Bayesian credible interval analysis with independent information from genomic, epigenomic, and spatial chromatin organization, we prioritize genes (including *IFIH1* and *IL23A*) that have implications for common molecular mechanisms involved in psoriasis and CAD inflammatory signaling. Chronic systemic inflammation has been associated with CAD and myocardial infarction, and Mendelian randomization analysis finds that CAD as an exposure can have a significant causal effect on psoriasis (*OR* = *1.11; p* = *3×10*^*−6*^) following adjustment for BMI and waist-hip ratio. Together, these findings suggest that systemic inflammation which causes CAD can increase the risk of psoriasis.

## Abstract (web summary)

Coronary artery disease (CAD) and psoriasis are established comorbidities, however their molecular relationship remains unclear. Here, the authors performed trans-disease meta-analysis, highlighting four genetic loci with evidence of colocalization, and prioritized genes based on multiomic data integration.

## Introduction

Psoriasis is a chronic inflammatory skin disease affecting ~3% of the adult US population^1^. In addition to its direct impact on patient health, psoriasis poses a substantial burden through its comorbidities^2,3^. Coronary artery disease (CAD) has evidence of increased incidence among psoriasis patients spanning more than 60 years^4–8^; it is the world’s leading cause of death with an estimated 197 million cases in 2019^9^. CAD subtly decreases blood supply to the heart through atherosclerosis such that a heart attack or sudden cardiac death is often the first symptom^10^. The risk of major adverse cardiac events is particularly high among patients with severe psoriasis (up to 1.42 hazard ratio)^11^, adjusting for other risk factors (including diabetes and hyperlipidemia). Furthermore, CAD has been found to be associated with Psoriasis Area and Severity Index (PASI)^12^; treatments which lower PASI can also improve coronary plaque^13–15^ and vascular inflammation^16^. The ACC/AHA guidelines for the prevention of cardiovascular disease list psoriasis as a risk-enhancing factor^17^, however until now there has been limited research to reveal the causal relationship between psoriasis and CAD.

Psoriatic patients exhibit systemic inflammation^18,19^, and studies have revealed that it can be induced by high BMI, infection, stress, drugs, etc., particularly for factors that have an impact on the immune system^20–22^. Psoriasis has a complex genetic profile, with most of the identified signals playing roles in immune-mediated pathways, including IL-23/NFκB/epidermal differentiation signaling^23–26^. The genetic architecture of CAD has also been studied extensively^27,28^, and findings highlight that CAD is associated with the dysregulation of metabolic and inflammatory interactions^29^. Mehta et al.^30^ found atherosclerosis-associated genes related to lipid metabolism to be downregulated in psoriasis skin while those that are inflammation-related were upregulated; it has also been posited that inflammation in psoriasis can lead to insulin resistance, which causes endothelial dysfunction and atherosclerosis^31^. Interestingly, lower levels of adiponectin have been observed in psoriasis patients, adjusting for cardiometabolic risk factors^32^, and it was speculated this may represent adipose inflammation. As a marker of systemic inflammation, C-reactive protein (CRP) has been proposed to predict the risk of CAD among psoriasis patients^33^. However, knocking out CRP in mice did not reduce atherosclerosis^34^, and a large-scale longitudinal study^35^ found CRP was not an independent predictor of plaque formation or progression. More work is therefore needed to pinpoint the molecular mechanisms which drive the association between CAD and psoriasis.

Here, we present a trans-disease meta-analysis (TDMA) to identify genetic loci that have shared or opposing effect between psoriasis and CAD in over 200,000 cases and controls. We prioritize the genes involved at these loci through a multi-omics approach, to help explain the molecular mechanisms CAD and psoriasis have in common. We also use Mendelian randomization to reveal a causal relationship between CAD and psoriasis independent of metabolic risk factors. Our findings illustrate the genetic risk factors which lead to the CAD/psoriasis comorbidity, improving understanding of which individuals are at greater risk and enabling advances in precision health care for them.

## Results

### Shared genetics/genomics

**Fig. 1:**
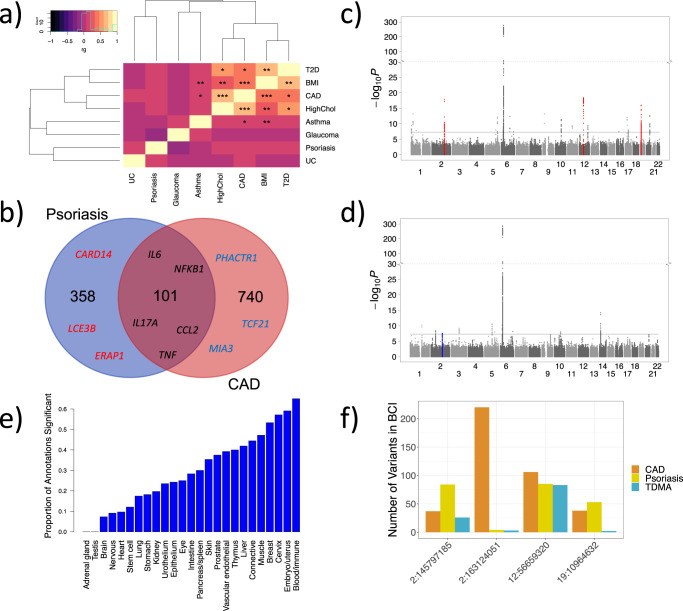
Shared genetic/genomic associations between psoriasis and CAD. **a** Heatmap of genetic correlations between psoriasis, CAD and six other traits, calculated by applying linkage disequilibrium score regression (LDSC) to summary statistics from the UK Biobank. The color scale indicates genetic correlation (r_g_), from strongly negative (purple) to strongly positive (yellow). *FDR < 0.01, **FDR < 1 × 10^−10^, ***FDR < 1 × 10^−20^. **b** Venn diagram illustrating the number of shared and distinct genes, with overall association score >0.1 in OpenTargets, involved in psoriasis and CAD. Examples of shared and distinct genes are overlaid; notably many of the shared genes are responsible for immune response. **c** Manhattan plot of shared (same direction of effect) psoriasis/CAD TDMA signals, showing markers that are more significant in TDMA than for either trait. The three loci we identified that are suggestive significant (*p* < 1 × 10^−4^) for both traits (meeting all our criteria) are highlighted in red. **d** Manhattan plot of opposing (opposite direction of effect) psoriasis/CAD TDMA signals, showing markers that are more significant in TDMA than for either trait. The locus we identified as suggestive significant (*p* < 1 × 10^−4^) for both traits (meeting all our criteria) are highlighted in blue. **e** Bar plot of the proportion of annotations, from GARFIELD enrichment analysis on TDMA outside the MHC, that are significant in each category. Notably, the Blood/Immune category has highest proportion of significant annotations, demonstrating an immunological basis for the shared genetics between psoriasis and CAD. **f** Grouped bar plot indicating the number of markers in 95% Bayesian credible intervals (BCI) calculated for psoriasis, CAD and TDMA at each locus. TDMA reduces the number of markers in the BCI for all for loci compared to either trait, thus facilitating improved fine-mapping.

Previous GWAS have identified more than 80 and 150 associated loci for psoriasis and CAD, respectively^36,37^. As a preliminary investigation of their genetic correlation, and to assess what can be achieved using existing datasets and approaches, we applied linkage disequilibrium score regression (LDSC)^38^ (a technique which estimates global correlation using summary statistics) to pre-prepared data from the UK Biobank^39^ (Supplementary Table 1). Figure 1a presents a heatmap and hierarchical clustering for the genetic correlations between psoriasis, CAD and their comorbidities. Notably, we identified a significantly correlated cluster of metabolic conditions including CAD, type 2 diabetes, body mass index (BMI), and high cholesterol. Despite its positive direction, the correlation between psoriasis and CAD was not significant (*r*_*g*_ = 0.14, *p* = 0.15). We further investigated the psoriasis/CAD genetic correlation by applying LDSC to our case-control psoriasis GWAS (11,024 cases and 16,336 controls) and a large CAD GWAS (60,801 cases and 123,504 controls), which provided a nominally significant (*p* = 0.02) genetic correlation (*r*_*g*_ = 0.11). Nevertheless, psoriasis and CAD have long been recognized as comorbidities^4–8^. Using electronic health records from 32,309 patients from the Michigan Genomics Initiative (MGI), we confirmed psoriasis and CAD co-occur more frequently than expected by chance (logistic regression (32,296 DF) *p* = 7.5 × 10^−5^, OR = 1.40, CI = 1.23–1.57), adjusting for age, gender, BMI, race, and socioeconomic disadvantage.

We then investigated a drug target database (the Open Targets platform^40^) to identify potential shared genes among the two conditions (using the genes reported by the platform to be associated with each condition, Methods). Figure 1b presents the number of shared and distinct disease-associated genes, indicating 101 out of 459 psoriasis genes (22%) are also involved in CAD (Supplementary Table 2). Interestingly, many of the shared genes relate to immune response. For example, *NFKB1* and *TNF* are involved in NF *κ*B signaling^41^, while *IL17A* is a key cytokine for psoriasis^42^. We compared the genes from Open Targets against a list experimentally determined in vivo by inflammatory stimulus on leukocytes from human blood^43^ and found that 45% of the shared genes had a role in systemic inflammation (Supplementary Table 3), as opposed to only 15% for the CAD-distinct genes and 26% for psoriasis-distinct genes (Fisher’s exact test *p* = 2.5 × 10^−8^, OR = 3.47, CI = 2.23–5.40). The shared gene architecture concords with clinical studies that show decreases in inflammation can benefit both diseases^15,44,45^; therefore, a better understanding of the intrinsic causal effects that lead to the disease comorbidities can provide further information about their shared mechanisms.

### Shared loci

**Fig. 2:**
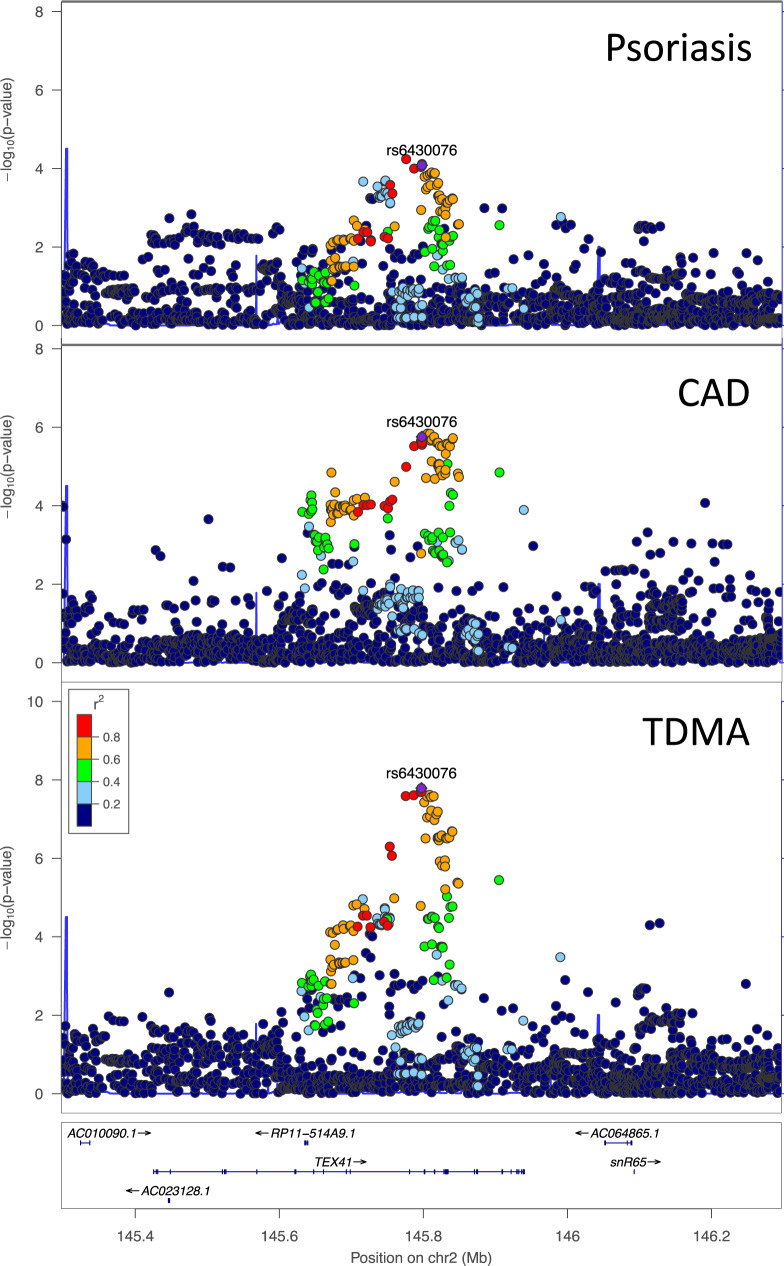
Regional association plots for psoriasis, CAD and TDMA at the chromosome 2 locus overlapping *TEX41*. The locus is suggestive significant for each disease and genome-wide significant in the trans-disease meta-analysis, with the lead marker from TDMA indicated in purple.

Applying trans-disease meta-analysis (TDMA)^46,47^ to the summary statistics for psoriasis and CAD, out of the 8,067,837 well-imputed markers common to both traits, 2,062,527 markers were more significant in the TDMA than the GWAS from either trait when testing for shared signals (i.e., allele exerting same effect for both diseases). Of the 44 genome-wide significant (*p* < 5 × 10^−8^) shared loci, three were identified as being at least suggestive significant in both traits (*p* < 1 × 10^−4^), with lead markers 2:163124051, 12:56659320, and 19:10964632, respectively (Fig. 1c). We also tested for opposing loci (with opposite direction of effect) and identified an additional locus with lead marker 2:145797185 (Fig. 1d). Details of the four loci identified by TDMA are provided in Table 1, along with their risk alleles and allele frequencies. For comparison, Supplementary Fig. 1 presents the signals from psoriasis and CAD GWAS. Figure 2 illustrates the opposing locus in more detail through regional association plots for psoriasis, CAD and TDMA. Notably, this locus is suggestive significant for each disease and genome-wide significant in trans-disease meta-analysis. The other three loci are shown in Supplementary Figs. 2–4.

**Table 1:** Trans-disease meta-analysis results for loci meeting criteria

Marker ID	Chr	Position (hg19)	Alleles (risk/non-risk)^b^	TDMA Direction	Meta-analysis *P*-values			Meta-analysis Odds ratios			Risk allele frequencies^a^		Nearby genes
					Psoriasis	CAD	TDMA	Psoriasis	CAD	TDMA	Psoriasis	CAD	
rs6430076	2	145797185	G/A	Opposing	8.2 × 10^−5^	1.8 × 10^−6^	1.6 × 10^−8^	0.92	1.05	1.07	0.35	0.32	*TEX41, ZEB2*
rs1990760	2	163124051	T/C	Shared	5.7 × 10^−15^	5.3 × 10^−5^	1.3 × 10^−18^	1.16	1.04	1.10	0.61	0.56	*IFIH1*
rs76151170	12	56659320	C/T	Shared	9.8 × 10^−16^	6.0 × 10^−5^	2.8 × 10^−19^	1.38	1.09	1.23	0.94	0.94	*IL23A. COQ10A*
rs12979495	19	10964632	G/A	Shared	1.3 × 10^−5^	4.7 × 10^−10^	2.5 × 10^−11^	1.10	1.07	1.08	0.74	0.75	*DNM2, ILF3*

*Chr* chromosome, *OR* odds ratio, *p* *p*-value, *CAD* coronary artery disease.

^a^Allele frequencies are provided for each entire meta-analysis (cases and controls), rather than specifically for each disease.

^b^Risk allele corresponds to TDMA. Gene names are italicized.

A recent trans-ethnic meta-analysis in CAD^48^ has confirmed signals in linkage disequilibrium (LD) with the opposing (*p* = 2.7 × 10^−14^, OR = 1.05, r^2^ = 0.69, D’ = 0.95) and the shared (*p* = 3.7 × 10^−8^, OR = 1.03, r^2^ = 0.89, D’ = 0.95) chromosome 2 loci (2:145797185 and 2:163124051) to be genome-wide significant. The opposing locus is also genome-wide significant for arterial stiffness in the UK Biobank^49^ (*p* = 5.3 × 10^−12^, OR = 1.03, r^2^ = 0.84, D’ = 1.00); while in addition to being a known psoriasis signal, the lead marker in the shared chromosome 2 locus (2:163124051) is a missense variant (predicted to be benign by SIFT^50^ and PolyPhen-2^51^, but having a high 15.24 CADD^52^ score) for *IFIH1*. Mutations in *IFIH1* are responsible for Singleton-Merten syndrome^53^ (a rare heart condition), Aicardi-Goutières syndrome^54^ (which can have skin symptoms) and susceptibility to viral respiratory infections^55^ (associated with both psoriasis^56^ and CAD^57^). The shared chromosome 2 lead marker (2:163124051) is associated with other immune-mediated conditions, such as vitiligo^58^ (*p* = 9.6 × 10^−20^, OR = 1.32), type 1 diabetes^59^ (*p* = 1.3 × 10^−17^, OR = 1.13), IgA deficiency^60^ (*p* = 3.7 × 10^−15^, OR = 1.43), autoimmune thyroid disease^61^ (*p* = 1.1 × 10^−14^, OR = 1.07), inflammatory bowel disease^62^ (*p* = 3.6 × 10^−10^, OR = 1.07), and systemic lupus erythematosus^63^ (*p* = 3.7 × 10^−8^, OR = 1.15). The chromosome 12 locus (12:56659320) is more strongly associated with psoriasis while the chromosome 19 locus (19:10964632) associates more with CAD, however both are negatively associated with height in the UK Biobank^38^ (*p* = 1.5 × 10^−18^, OR = 0.97 and *p* = 8.4 × 10^−14^, OR = 0.99). Shorter height is believed to increase the risk of CAD^64^, potentially because of its impact on adipose distribution. Interestingly, none of the four loci we identified are significantly associated with BMI^65^ or waist-hip ratio (WHR)^66^, while only the chromosome 19 lead marker is associated with LDL (*p* = 3 × 10^−26^, OR = 1.06).

To confirm the TDMA loci have the same causal signal, we performed colocalization analysis using COLOC^67^. Three of the four loci had a high posterior probability (*PP*) of having the same causal variant: *PP* = 0.86 for the opposing chromosome 2 locus (2:145797185); *PP* = 0.89 for the shared chromosome 2 locus (2:163124051); and *PP* = 0.85 for the shared chromosome 12 locus (12:56659320). The chromosome 19 locus (19:10964632) had a higher posterior probability of there being two independent signals for CAD and psoriasis (*PP* = 0.55), as opposed to a shared causal signal (*PP* = 0.45). To investigate this further, we applied the Sum of Single Effects (SuSiE) COLOC extension^68^, which uses fine-mapping to perform more accurate inference for locus with multiple causal variants, and this gave a posterior probability of 1.0 for the signal identified by TDMA being shared. We also used multi-trait, conditional and joint analysis (mtCOJO)^69^ in GCTA to adjust for BMI^66^ on the CAD summary statistics and found the colocalizations still remained.

### Functional analysis

We investigated the functional impact of the shared psoriasis/CAD genetic signals, focusing on 2,054,337 non-MHC markers that are more significant in TDMA than either disease, by applying GARFIELD^70^, a powerful enrichment tool which compares 997 different sets of annotation marks from specific cell types, adjusting for LD, MAF and distance to transcription start site. Chromatin marks in blood were particularly enriched (Supplementary Fig. 5), with over 100 significant annotations, after adjusting for Bonferroni correction. However, blood tissue also has the most annotations (166 in total) and some of the tissue types are closely related (for example intestine and colon, or cerebellar and brain), so to achieve a fair comparison (adjusting for the number of annotations from each tissue), we combined related tissues together into 26 categories, and then studied the proportion of significant annotations for each category (Fig. 1e). Notably, the blood/immune category (which comprises only blood tissue) had the highest proportion of significant annotations (65%). Furthermore, of the other highly enriched categories, cervix (57% significant) is dominated by HeLa-S3 (a cancer cell line) and the embryo/uterus tissue (59% significant) involves multiple immune mechanisms^71^. Interestingly, normal skin and heart tissue had moderate to low enrichment (35% and 10% significant, respectively), further suggesting that systemic inflammation rather than tissue-specific mechanisms are responsible for the shared signals. In addition, when we used our functional enrichment analysis approach (MEAGA)^72^ to study the functions of genes that are close to (within 100k of) CAD loci but are not associated with psoriasis (*p* > 1 × 10^−4^ in psoriasis GWAS), the most significant functions are only associated with metabolic behaviors, including lipid transport (*p* < 5 × 10^−4^), which is with high contrast with the strong immunological functions that are associated with psoriasis loci^24^. These results highlight the involvement of both metabolic and immunological pathways for the CAD susceptibility loci.

### Gene prioritization

**Fig. 3:**
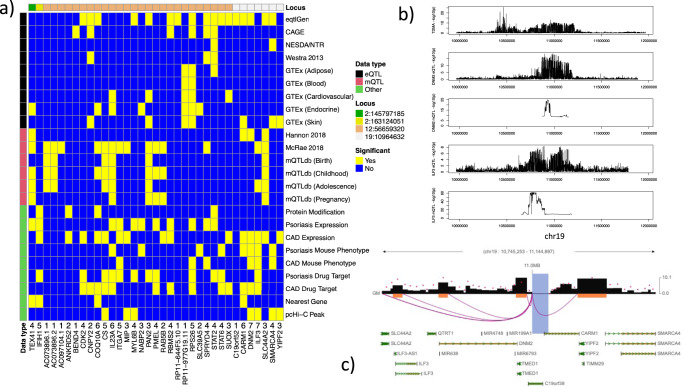
Gene prioritization. **a** Matrix of candidate genes by supporting evidence. Genes are included as candidates if one of the markers in the 95% Bayesian credible interval (BCI) set for each TDMA locus is a significant eQTL, mQTL or have a direct protein modification effect for that gene. Cells colored in yellow indicate genes with significant supporting evidence, while cells colored in blue do not. Sources of supporting evidence are grouped into three categories: eQTL (black), mQTL (red), and other (**green**). The color bar at the top of the matrix indicates the locus each gene belongs to (ordered by genomic position), with the position of the TMDA lead marker for each locus being indicated in the legend. The row of numbers, below the matrix and above the gene names, is a score, calculated by adding up the number of sources of evidence for that gene and counting the presence of any eQTL or mQTL only once, respectively, to avoid biasing towards these correlated data sources. **b** Regional association plots comparing TDMA genetic signals at the chromosome 19 locus with eQTL and mQTL signals (from eQTLGen and Hannon et al., respectively) for the two highest scoring genes (*DNM2* and *ILF3*). **c** Promoter-capture Hi-C interactions for the chromosome 19 locus in lymphoblastoid cells. Shaded regions represent the extent of the interaction contacts. The region which encompasses the lead marker from TDMA interacts with promoters for *DNM2* and *ILF3*.

For each of the four loci identified by TDMA, we calculated 95% Bayesian credible intervals (BCI) using the psoriasis/CAD summary statistics as well as the appropriate TDMA statistics (shared vs. opposing direction). Figure 1f presents the number of markers in each BCI set, which is consistently lower for TDMA than either trait, suggesting it could facilitate the fine-mapping of causal variants. To prioritize the genes for each locus, we constructed a matrix of available evidence (Fig. 3a), including gene candidates that have a significant eQTL or methylation-QTL, mQTL (*p* < 1 × 10^−6^) or a direct protein modification corresponding to one of the markers in the BCI for each locus. In addition, we considered whether the genes are differentially expressed in psoriasis skin or CAD peripheral blood compared to controls, are associated with mouse phenotypes enriched for psoriasis/CAD, are targets for psoriasis/CAD drugs, are the nearest gene to the lead marker or have a significant pcHi-C contact in lymphoblastoid cells. For each candidate gene, we calculated an aggregate score by adding up the number of sources of evidence for that gene. These scores range from 1 for the least through to 7 for the most supporting evidence.

The chromosome 2 shared (2:163124051) and opposing (2:145797185) loci each only had one candidate gene that met our criteria, *IFIH1* (score: 5) and *TEX41* (score: 4). Previous research^49^ has also suggested *ZEB2* as a candidate^49,73^ near the opposing locus and GTEx has a suggestive significant aorta eQTL for this gene in the BCI set (rs1881410, *p* = 4.4 × 10^−6^). As with *TEX41* (*FC* = 0.41*, p* = 1.4 × 10^−5^), *ZEB2* (*FC* = 0.42*, p* = 2.8 × 10^−12^) is significantly downregulated in psoriasis, however there was no mQTL or other evidence for this gene. The chromosome 12 locus (12:56659320) had the most candidate genes (26 as opposed to 7 for chromosome 19 (19:10964632)). Of these, *COQ10A* and *IL23A* had the highest prioritization, each with an aggregate score of 6. *COQ10A* is the nearest gene to the lead marker for this locus, and one of the markers in the BCI set (rs60542959) is a start lost variant for this gene (although it is predicted to be benign by SIFT and PolyPhen-2). *COQ10A* is upregulated in CAD (FC = 3.76, *p* = 2.4 × 10^−3^), while *IL23A* is upregulated in psoriasis (FC = 6.91, *p* = 1.8 × 10^−10^). Both are CAD drug targets, although *IL23A* is also a drug target for psoriasis, and both have eQTL and mQTL evidence.

Figure 3b shows eQTL (from eQTLGen) and mQTL (from Hannon et al.^74^) signals for the two highest ranking genes in chromosome 19 (19:10964632), as regional association plots against the TDMA genetic signals for this region. *DNM2* and *ILF3* both have eQTL signals which match more closely with the TDMA locus than the psoriasis-specific locus upstream. To test for causal as opposed to pleiotropic associations, we applied Summary-based Mendelian Randomization (SMR) to the eQTL and TDMA summary statistics and found both genes to have significant causal effect on both traits (*DNM2*: *p* = 1.1 × 10^−6^; *ILF3*: *p* = 1.4 × 10^−5^). Furthermore, heterogeneity in dependent instruments (HEIDI) tests showed no significant issues with linkage (*DNM2*: p = 0.17; *ILF3*: *p* = 0.55). SMR results for the mQTLs were also significant (*DNM2*: *p* = 2.6 × 10^−6^; *ILF3*: *p* = 1.1 × 10^−5^), however HEIDI suggests some potential heterogeneity in the causal estimates (*DNM2*: *p* = 2.1 × 10^−3^; *ILF3*: 2.0 × 10^−2^). Notably, the mQTL signal for *DNM2* is much narrower than the eQTL or TDMA signal, and the mQTL signal for *ILF3* aligns only with the upstream part of the eQTL signal. Nevertheless, promoter-capture Hi-C analysis in lymphoblastoid cells (Fig. 3c) demonstrates significant interactions between the chromosome 19 TDMA locus (19:10964632) and both of the promoters for *DNM2* and *ILF3*.

### Mendelian randomization

Our TDMA signals are enriched for immune cells as opposed to skin or heart tissue, and none of the loci we identified are associated with BMI. To assess whether there is a potential causal relationship between psoriasis and CAD, independent of other modifiable risk factors, we applied Mendelian randomization (MR), a technique that uses genetic markers as instruments to test for causality in a way that is robust to confounding factors. We used three standard MR techniques, inverse-variance weighted (IVW), median and mode, in addition to a more advanced technique, MR-RAPS^75^, which controls for pleiotropy through a random effect model and takes into account the variance in instrument effect sizes. IVW, median and MR-RAPS approaches consistently identified CAD as having a significant causal effect on psoriasis (*p* = 2.2 × 10^−3^ for MR-RAPS), with comparable effect size (from OR = 1.13 for MR-RAPS to OR = 1.16 for median MR). The effect size estimated by mode MR was even larger (OR = 1.45), but its p-value was only nominally significant (*p* = 0.02). By contrast, none of the approaches found psoriasis to have a causal effect on CAD. A further approach, MR-Egger^76^, which controls for pleiotropy by including the intercept in its model, did not find CAD to have a causal effect on psoriasis (*p* = 0.50, OR = 1.05). We then excluded the four loci identified by TDMA as potential sources of this pleiotropy and repeated the MR analysis. Again, the IVW, median and MR-RAPS approaches confirmed a consistent causal effect (*p* = 4.6 × 10^−3^, OR = 1.11 for MR-RAPS), while MR-Egger did not (*p* = 0.61, OR = 1.04). Similarly, excluding all known psoriasis loci was still significant for CAD on psoriasis (*p* = 8.7 × 10^−3^, OR = 1.10) in MR-RAPS.

We considered the following potential confounders for causal inference: BMI, WHR, type 2 diabetes (T2D), HDL cholesterol, LDL cholesterol, total cholesterol (TC) and triglycerides (TG). Testing each trait one at a time (Table 2), revealed BMI (*p* = 1.5 × 10^−9^, OR = 1.48 for MR-RAPS) and WHR (*p* = 1.0 × 10^−6^, OR = 1.53 for MR-RAPS) to have a significant causal effect on psoriasis, consistently across the approaches except mode MR. While, for CAD, all seven metabolic conditions had a significant causal effect (only BMI, T2D and TC were significant in mode MR). We applied multivariable MR (GRAPPLE^77^) to control for BMI and WHR, and the effect of CAD on psoriasis was consistent (*p* = 3.1 × 10^−6^, OR = 1.11), while BMI (*p* = 1.9 × 10^−3^, OR = 1.28) and WHR (*p* = 2.8 × 10^−2^, OR = 1.23) had a reduced effect size. Excluding WHR (the least significant of these two traits) gave comparable effect size to the univariable analysis (CAD: *p* = 5.3 × 10^−3^, OR = 1.10; BMI: *p* = 5.3 × 10^−7^, OR = 1.40). The causal effects of CAD on psoriasis still hold even after removing all markers from psoriasis-associated loci. These results therefore suggest that CAD has a causal effect on psoriasis that is independent of the above potential metabolic confounders.

**Table 2:** Results for Mendelian randomization

	Num. markers	Psoriasis								CAD							
		IVW		Median		Mode		MR-RAPS		IVW		Median		Mode		MR-RAPS	
		OR	*P*	OR	*P*	OR	*P*	OR	*P*	OR	*P*	OR	*P*	OR	*P*	*OR*	*P*
**Psoriasis**	184	—	—	—	—	—	—	—	—	1.01	0.14	1.00	0.64	1.00	0.91	1.01	0.09
**CAD**	448	**1.14**	**6.3** **×** **10**^**−4**^	**1.16**	**3.6** **×** **10**^**−3**^	1.45	0.02	**1.13**	**2.2** **×** **10**^**−3**^	—	—	—	—	—	—	—	—
**BMI**	969	**1.48**	**1.5** **×** **10**^**−9**^	**1.38**	**1.0** **×** **10**^**−3**^	1.96	0.08	**1.50**	**2.2** **×** **10**^**−9**^	**1.51**	**5.1** **×** **10**^**−71**^	**1.52**	**1.6** **×** **10**^**−61**^	**1.45**	**2.8** **×** **10**^**−4**^	**1.51**	**1.6** **×** **10**^**−69**^
**WHR**	796	**1.53**	**1.0** **×** **10**^**−6**^	**1.49**	**4.4** **×** **10**^**−4**^	1.12	0.78	**1.52**	**3.1** **×** **10**^**−6**^	**1.56**	**4.5** **×** **10**^**−50**^	**1.60**	**5.9** **×** **10**^**−47**^	1.23	0.21	**1.57**	**3.6** **×** **10**^**−50**^
**T2D**	561	1.05	0.08	1.07	0.08	0.86	0.29	1.06	0.06	**1.16**	**1.1** **×** **10**^**−41**^	**1.18**	**1.4** **×** **10**^**−45**^	**1.16**	**4.3** **×** **10**^**−4**^	**1.16**	**3.3** **×** **10**^**−39**^
**HDL**	362	1.04	0.44	0.92	0.35	1.23	0.21	1.06	0.27	**0.87**	**6.1** **×** **10**^**−9**^	**0.87**	**1.5** **×** **10**^**−8**^	0.89	0.17	**0.88**	**9.6** **×** **10**^**−11**^
**LDL**	301	1.04	0.43	0.98	0.78	0.86	0.29	1.02	0.67	**1.31**	**7.2** **×** **10**^**−35**^	**1.19**	**2.3** **×** **10**^**−13**^	1.11	0.19	**1.44**	**3.4** **×** **10**^**−211**^
**TC**	308	1.12	0.03	1.03	0.63	0.97	0.85	1.12	0.04	**1.38**	**7.4** **×** **10**^**−41**^	**1.22**	**3.9** **×** **10**^**−16**^	**1.43**	**8.6** **×** **10**^**−4**^	**1.48**	**1.4** **×** **10**^**−222**^
**TG**	353	1.07	0.16	1.01	0.87	0.84	0.34	1.06	0.22	**1.21**	**3.6** **×** **10**^**−20**^	**1.16**	**2.7** **×** **10**^**−10**^	1.15	0.02	**1.21**	**5.8** **×** **10**^**−24**^

Results significant after Bonferroni adjustment are indicated in bold.

*IVW* inverse-variance weighted, *Num*. number of, *CAD* coronary artery disease, *BMI* body mass index, *WHR* waist-hip ratio, *T2D* type 2 diabetes, *HDL* high-density lipoprotein, *LDL* low-density lipoprotein, *TC* total cholesterol, *TG* triglycerides, *OR* odds ratio, *p* *p*-value.

Finally, we applied logistic regression to study the effect of hyperlipidemia (which leads to CAD) among 225 psoriasis patients from our cohort, for which the health survey was taken within two years of psoriasis onset. Compared to 2,457 unaffected controls, the patients with recent psoriasis diagnosis had a significantly higher rate of hyperlipidemia (OR = 1.58, *p* = 4.8 × 10^−3^) independent of BMI (OR = 1.41, *p* = 3.4 × 10^−8^) and age (OR = 1.24, *p* = 8.0 × 10^−3^), suggesting the conditions leading to CAD may occur before psoriasis begins. We then modeled the early psoriasis onset (young onset of psoriasis ~ BMI + hyperlipidemia + recent onset of psoriasis + hyperlipidemia * recent onset of psoriasis) among 2212 psoriasis patients (of which 1062 had age at onset ≤25-year). Hyperlipidemia was negatively associated with young age at onset (OR = 0.64, *p* = 1.2 × 10^−6^). More interestingly, its interaction effect with recent (≤2 years) onset was also negative (OR = 0.13, *p* = 5.7 × 10^−3^), after controlling for BMI. Since patients with hyperlipidemia and a recent psoriasis onset could indicate the hyperlipidemia was developed before psoriasis, our results show that patients with older age at onset tend to have hyperlipidemia before psoriasis, suggesting the conditions surrounding hyperlipidemia (leading to CAD) can trigger psoriasis. This is in agreement with a recent study^78^, which showed that late onset psoriasis is more often associated with metabolic disease.

## Discussion

Our trans-disease meta-analysis revealed BMI-independent loci that are shared between psoriasis and CAD. The three shared loci (with lead markers 2:163124051, 12:56659320, and 19:10964632) as well as an opposing locus (2:145797185) identified by TDMA were further confirmed through colocalization (even after controlling for BMI). Using enrichment analysis, we showed that the shared signals are largely driven by immune components. Importantly, the Bayesian credible interval sets for TDMA were consistently smaller than for either trait, which allowed us to pinpoint the genes involved at each locus, through a combination of eQTLs, mQTLs, protein modifications, expression data, mouse phenotypes, drug targets, and promoter-capture Hi-C.

Mendelian randomization (MR) suggests the relationship we identified between psoriasis and CAD has a causal basis, independent of BMI and WHR. This finding is particularly interesting as it differs from our prior expectation that systemic inflammation caused by psoriasis leads to CAD. However, it is known that subclinical atherosclerosis can exist for many years, as an inflammatory process^79,80^, before any symptoms become apparent^10^; underlying inflammatory conditions may trigger psoriasis, as was suggested by MR studies connecting obesity to psoriasis^22,81^. Bagchi et al.^82^ found that ApoE-deficient mice (that have elevated lipid levels) develop psoriasis-like lesions in the presence of CD1b-autoreactive T cells. Although we did not find any causal effect of lipid traits on psoriasis in our MR, we did observe their effect on CAD, which is consistent with hyperlipidemia being a known risk factor^10^. The age of onset for psoriasis is highly variable, and can occur at any time from birth to the eighth or ninth decade^83^. Our results suggest that hyperlipidemia, a known causative risk factor for the development of CAD, promotes older age-onset psoriasis. On the contrary, the findings of previous population based studies also show that young patients with psoriasis have a higher relative risk of myocardial infarction^7,84^ and adverse cardiovascular events^85^, though the causal relationship is unknown. Future study is required to better understand the potential differences in association and effect between CAD versus young/old-onset of psoriasis.

Previous studies report limited genetic overlap between psoriasis and CAD. Gupta et al.^86^ examined a catalog summarizing published GWAS results and concluded the conditions are more likely to be linked by environmental factors, such as diet. Koch et al.^87^ assessed the genetic overlap in a German health system and found no significant signals outside the MHC. Compared with these studies, our analysis involved much larger sample sizes (11,024 psoriasis and 60,801 CAD patients, along with accompanying controls) and we applied an innovative trans-disease meta-analysis approach involving strict criteria to confirm genetic loci as being shared. More recently, Rakhshan et al. suggested (based on analysis in 286 psoriasis patients and 300 controls) that CAD-associated genetic variants in *CDKN2B-AS* may also be associated with psoriasis, however in our own psoriasis GWAS we found no significance for these markers (even at the nominal level).

Our work has some limitations. When prioritizing gene candidates for each locus, we included GTEx eQTLs from adipose, blood, cardiovascular, endocrine, and skin tissue, along with an additional four eQTL studies from blood. Due to lack of datasets from other tissues, all the mQTL studies we included are blood based, and while important for interpretation of immunological processes, they may have some limitation in regards to our results involving inflammation in more complex tissues. In addition, the genetic associations depend on the number of cases and controls in each data source, and the statistical power that they provide. Furthermore, the LDSC correlation between psoriasis and CAD is dependent upon large GWAS meta-analyses, and not observed in smaller cohorts such as the UK Biobank. However, the signals we identified in TDMA all had the same direction of effect for psoriasis and CAD in a PheWAS of the UK Biobank^88^, and in most cases were at least nominally significant (Supplementary Table 4). Lastly, psoriasis and CAD are both immune-mediated diseases with genetic signals in the MHC, however we excluded this region due to the highly complex LD patterns, which can complicate the identification of specific signals. Future work, which is beyond the scope of the current study, will be needed to apply advanced HLA typing techniques to each disease to assess whether there are shared or opposing alleles in the MHC region.

Epidemiological results can be affected by the sensitivity and specificity of the ICD-9/10 codes used to select patients. It has been estimated that 81% of individuals with an ICD-10 code for psoriasis have a confirmed diagnosis^89^, while for ICD-9, the sensitivity is 88%^90^. A study using ICD-9 codes found 57% sensitivity and 96% specificity for CAD, although broader codes were used than in our study^91^; as far as the authors are aware, there have been no similar investigations for CAD using ICD-10 codes. Mendelian randomization depends on three key assumptions: that the instruments (genetic markers) associate with the exposure (risk factor), that they only affect the outcome through the exposure, and that they are not associated with any confounder. We addressed the first assumption by requiring genetic markers to have at least *p* ≤ 1 × 10^−4^ in the GWAS for each exposure, and applied MR-RAPS^75^ to control for any weak instruments bias. MR-RAPS accounts for potential pleiotropy (second assumption) using a random effects model, and we also applied the more traditional MR-Egger, which tests for pleiotropy by including the intercept. The third assumption was addressed by including potential confounders in multivariable analysis (MR-GRAPPLE), and we also repeated our analysis using alternative GWAS for CAD^27^ and lipids^92^, finding a consistently significant causal effect of CAD on psoriasis (*p* = 1.0 × 10^−3^, OR = 1.12) controlling for confounders.

Psoriasis and CAD are however known to share certain cytokines, including IFNγ, TNF, IL-6, and IL-17^93,94^. In addition to its well defined role in psoriasis^95^, IL-17 is produced by coronary artery-infiltrating T cells^96^ and atherosclerotic plaques^97^; Blocking IL-17A reduces atherosclerosis in mice^98,99^. *IL23A* (one of the highest prioritized genes for chromosome 12) interacts extensively with IL-17^100^; the other gene with highest prioritization score, *COQ10A*, has been found to inhibit IL-17 signaling in mice^101^ and also to modulate TNF^102,103^. Variants in *IFIH1*, the gene nominated for the chromosome 2 shared locus, are associated with IL-6 in systemic lupus erythematosus^104^. *ILF3*, one of the genes with highest score in chromosome 19, is necessary for the Il-2 expression in T cells^105^, which in turn induces IFNγ^106,107^; Conditional knockout experiments in mice have shown *ILF3* to have a key role in atherosclerotic calcification^108^. The involvement of these cytokines can have important implications for treatment. Consistent with our findings, psoriasis patients taking TNF inhibitors have been found to have less adverse cardiovascular events^109^ and subclinical atherosclerosis^110^. It should also be noted that CoQ_10_ can be used as a supplement and, in patients with acute coronary syndrome, was found to reduce ACE^111^, which is increased in psoriasis and associated with carotid intima-media thickness^112^.

Overall, our study has helped to identify four psoriasis/CAD shared and opposing genetic loci, each with candidate genes that provide valuable information on molecular mechanisms and potential treatment options. Most importantly, we have shown that the inflammation involved in subclinical atherosclerosis can have a causal effect on psoriasis.

## Methods

### Genetic correlations

Genetic correlations were estimated by applying linkage disequilibrium score regression (LDSC)^38^ to summary statistics from the UK Biobank, prepared by the Neale lab^113^. All the data used in LDSC was self-reported, except BMI which was measured at the initial assessment center visit. Hierarchical clustering was performed using heatmap.2 under default parameters. We also applied LDSC to summary statistics from physician-diagnosed psoriasis and CAD GWAS meta-analyses, using the data collection, processing and quality control steps described for each trait^23,114^.

### Epidemiology

Individuals were included from the Michigan Genomics Initiative and extracted using the University of Michigan’s DataDirect^115^ that have at least one health system encounter between January 1, 2019 and June 20, 2020, with race, age, gender, body mass index (BMI) and socioeconomic disadvantage recorded (as covariates). Patients were considered to have psoriasis that are annotated with ICD-9 691.0 or 696.1, or any of the L40 ICD-10 subcodes; patients were considered to have CAD that are annotated with any of the 414 ICD-9 subcodes, or I25 ICD-10 subcodes. Socioeconomic disadvantage was estimated by a DataDirect filter on the 2013–2017 American Community Survey (ACS) and obesity was graded into three categories (BMI < 30, 30–35, 35–40, ≥40)^47^. Analysis was performed using a logistic regression model. The study was approved by the University of Michigan institutional review board.

### Trans-disease meta-analysis

We performed trans-disease meta-analysis (TDMA) on the psoriasis and CAD GWAS meta-analyses using an equally weighted combination of the effect sizes (${\beta }_{{{{{{\rm{CAD}}}}}},{{{{{\rm{PsV}}}}}}}=\frac{{\beta }_{{{{{{\rm{CAD}}}}}}}+{\beta }_{{{{{{\rm{PsV}}}}}}}}{2}$ for shared effects, and ${\beta }_{{{{{{\rm{CAD}}}}}},{{{{{\rm{PsV}}}}}}}=\frac{{\beta }_{{{{{{\rm{CAD}}}}}}}-{\beta }_{{{{{{\rm{PsV}}}}}}}}{2}$ for opposing) and variances (${V}_{{{{{{\rm{CAD}}}}}},{{{{{\rm{PsV}}}}}}}=\frac{{V}_{{{{{{\rm{CAD}}}}}}}+{V}_{{{{{{\rm{PsV}}}}}}}}{4}$). Loci were identified that are at least 500 kb apart and only considered to be shared/opposing if they meet the following three criteria: (i) genome-wide significant (*p* < *5×10*^*−8*^) in TDMA; (ii) suggestive significant (*p* < 1 × 10^−4^) for each individual trait; and (iii) more significant in TDMA than both individual traits.

### Colocalization

We performed colocalization analysis on the four TDMA loci by applying COLOC^67^ to the psoriasis and CAD summary statistics, including all markers within ±100 kb of the lead TDMA marker. We also used the SuSiE extension^68^, available within the same library, which performs fine-mapping before colocalization. COLOC-SuSiE requires a matrix of signed linkage disequilibrium (r) values, which we generated from the largest single cohort of our psoriasis GWAS (11,675 individuals, cases and controls) using PLINK 1.9^116^. To ensure our results were not affected by BMI, we conditioned the CAD summary statistics on BMI by applying mtCOJO^117^ in GCTA with BMI summary statistics from 806,834 individuals in the GIANT consortium^66^, using the same LD information as a reference, and then repeated the colocalization analysis.

### Gene prioritization

We combined multiple sources of information to prioritize the genes for each locus. Five cis-eQTL datasets: one in whole blood (eQTLGen^118^), three in peripheral blood (CAGE^119^, NESDA/NTR^120^, and Westra et al.^121^), and one in multiple tissues (GTeX v8^122^); and three mQTL datasets: two in whole blood (Hannon et al.^74^, McRae et al.^123^) and one in cord and peripheral blood from multiple age groups (mQTLdb^124^) were used to select candidate genes from markers in the 95% BCI sets for TDMA;. for consistency, we only considered signals *p* < 1 × 10^−6^ as significant, matching the most stringent threshold (from CAGE). Genes were also included if they have a protein modifying variant from the BCI, annotated in Ensembl^125^. For each candidate gene, we calculated an overall score by adding up the number of sources of evidence. However, we only count the presence of any eQTL or mQTL once, to avoid biasing our prioritization towards these correlated data sources

We identified candidate genes as differentially expressed in psoriasis^126^ (28 lesional skin and 38 controls) and/or CAD^127^ (6 cases and 9 controls from peripheral blood). Mouse phenotypes were extracted from the Mouse Genome Informatics database^128^ and enrichment performed for psoriasis and CAD genes with an overall score ≥0.3 in Open Targets^40^, identifying two phenotypes each for psoriasis (MP:0005381 and MP:0005388) and CAD (MP:0005369 and MP:0005385) after Bonferroni correction. Gene targets for psoriasis and CAD drugs were identified by combining multiple resources in our previous work^129^; we only considered drugs annotated as being used for psoriasis/CAD in more than one dataset and genes targeted by more than one drug. The nearest gene for each locus was identified by measuring the distance (in base pairs) to the transcription start site. Lymphoblastoid promoter-capture Hi-C contacts were identified in the 3D-genome Interaction Viewer (3DIV)^130^, under the default *p* < *0.01* threshold.

Summary Mendelian randomization (SMR) and heterogeneity in dependent instruments (HEIDI) analysis^69^ were performed for eQTL/mQTL signals within ±1Mbp of the lead TDMA marker from each locus, using the default *p* ≤ 5 × 10^−8^ for SMR and *p* ≤ 1.57 × 10^−3^ for HEIDI, with data from the Haplotype Reference Consortium^131^ as the reference panel.

### Mendelian randomization

Mendelian randomization was performed on the psoriasis and CAD GWAS meta-analyses using TwoSampleMR^132^ for univariable analysis and GRAPPLE^77^ for multivariable analysis. In addition to the aforementioned psoriasis, CAD and BMI summary statistics, we included GWAS meta-analyses of waist-hip ratio (WHR) from 697,734 individuals in the GIANT consortium^66^, as well as HDL cholesterol, LDL cholesterol, total cholesterol and triglycerides from 94,674 individuals in the Kaiser Permanente health system^133^. Markers were selected as genetic instruments for each trait through linkage disequilibrium (LD) clumping in PLINK 1.9^116^ markers *p* ≤ 1 × 10^−4^ that are in the intersection of markers across traits, using the 1000 Genomes European samples (LD ≥ 0.001, window size = 10 Mbp). In multivariable analysis, we pooled the genetic instruments from each exposure.

### Reporting summary

Further information on research design is available in the Nature Research Reporting Summary linked to this article.

## Supplementary information

Supplementary Information

Reporting Summary

## Data Availability

Summary statistics for the coronary artery disease GWAS are available online (http://www.cardiogramplusc4d.org), along with the BMI and waist-hip ratio GWAS (https://portals.broadinstitute.org/collaboration/giant/index.php/GIANT_consortium), and the LDSC-formatted UK Biobank summary statistics (http://www.nealelab.is/uk-biobank). HDL cholesterol (GCST007140), LDL cholesterol (GCST007141), total cholesterol (GCST007143), and triglyceride (GCST007142). GWAS summary statistics are available from the European Bioinformatics Institute. Data for three of the psoriasis cohorts (phs000019.v1.p1; phs001306.v1.p1; phs000982.v1.p1) are available in dbGap, with data for the other cohorts available upon reasonable request. Access to data from the Michigan Genomics Initiative can be requested through the JIRA ticketing system (https://precisionhealth.umich.edu/our-research/michigangenomics/). eQTL data from eQTLGen (https://www.eqtlgen.org), CAGE (https://shiny.cnsgenomics.com/CAGE/), NESDA/NTR (https://eqtl.onderzoek.io/), Westra et al. (https://genenetwork.nl/bloodeqtlbrowser/) and GTeX (https://gtexportal.org/), as well as the mQTL datasets Hannon et al. and McRae et al. (https://cnsgenomics.com/software/smr/#DataResource) and mQTLdb (http://www.mqtldb.org) are available online. Expression data for psoriasis (GSE121212) and CAD (GSE23561) are available through GEO. The Mouse Genome Informatics database is available online (http://www.informatics.jax.org), as is the 3D-genome Interaction Viewer (http://kobic.kr/3div).
