# Supplementary material for: Shared genetic risk factors and causal association between psoriasis and coronary artery disease
Source: Nat Commun. 2022 Nov 2;13:6565. doi: 10.1038/s41467-022-34323-4 (PMC9630428; doi:10.1038/s41467-022-34323-4)
Supplement: Supplementary file 1 — Supplementary Information [file 41467_2022_34323_MOESM1_ESM.pdf]

Psoriasis

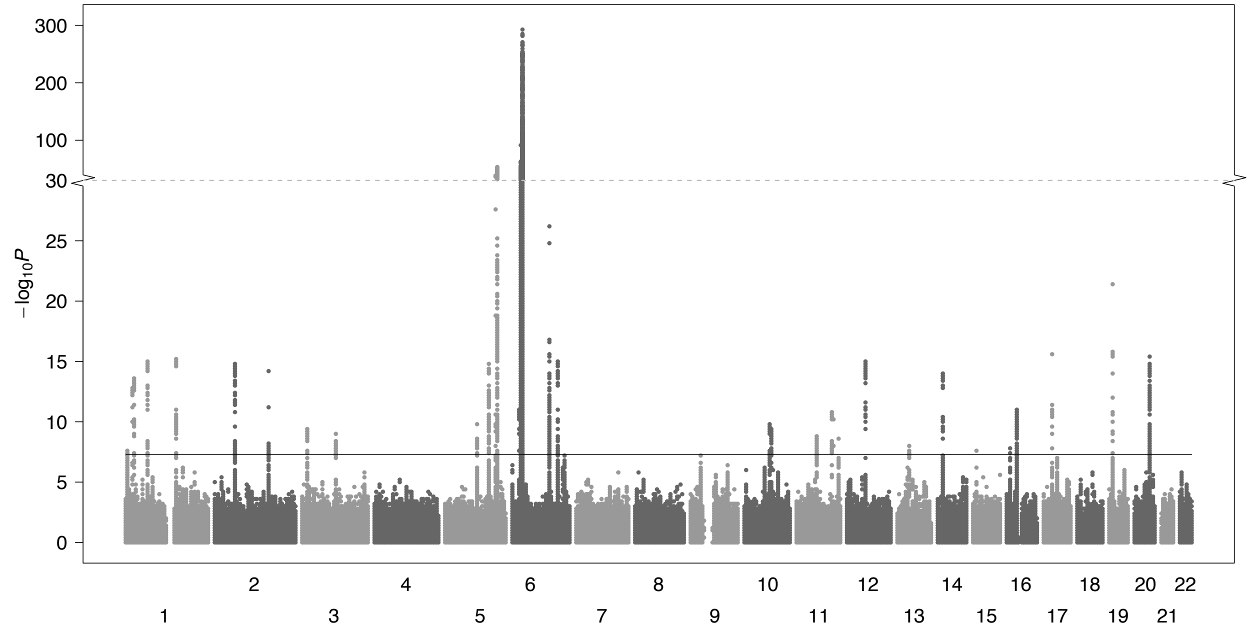

CAD

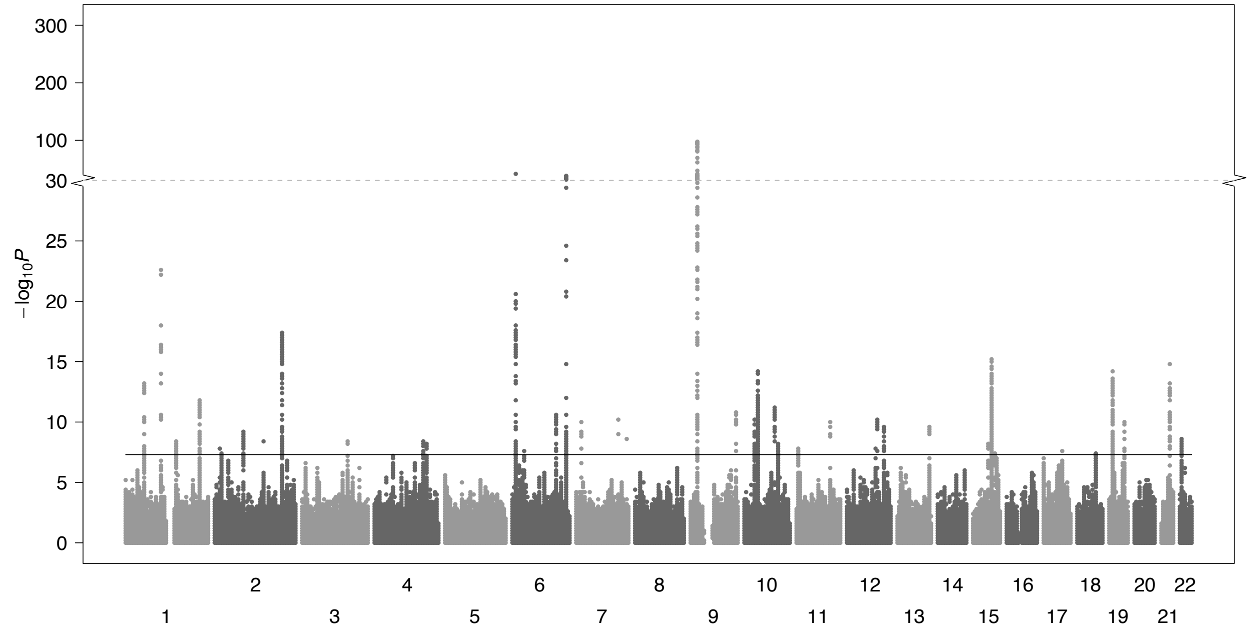

Supplementary Figure 1: Manhattan plots for psoriasis and CAD GWAS signals.

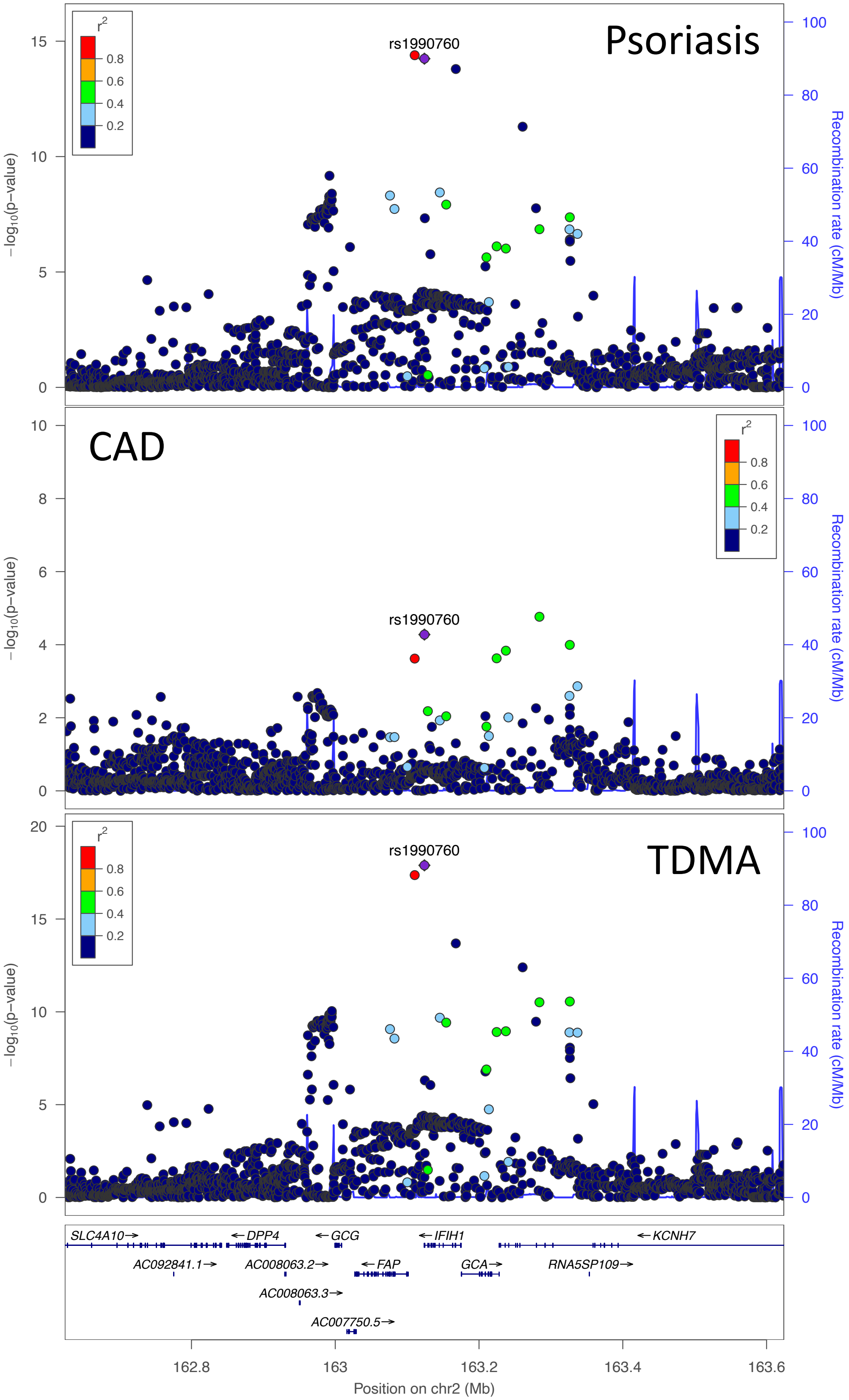

Supplementary Figure 2: Regional association plots for shared locus in chromosome 2.

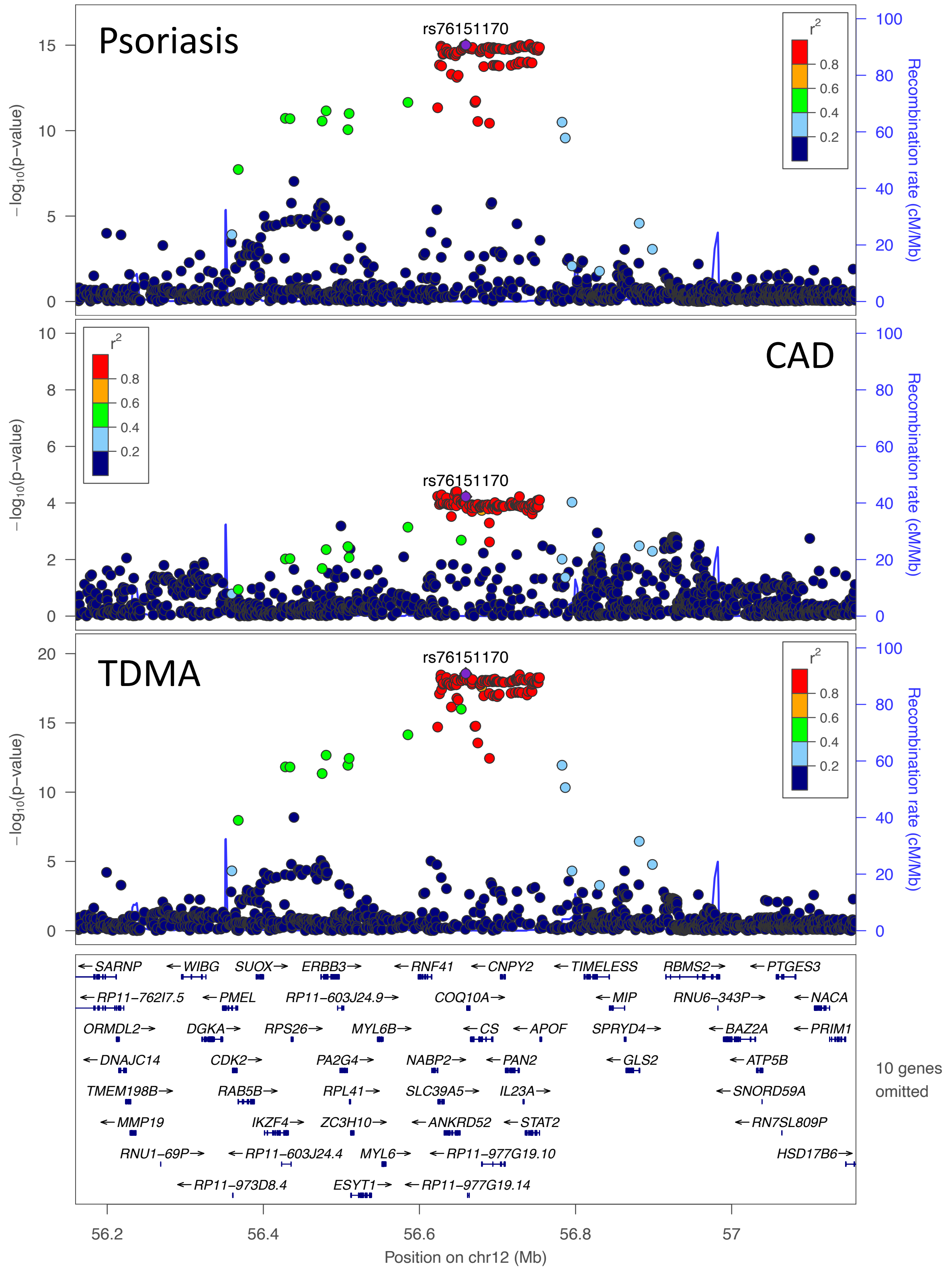

Supplementary Figure 3: Regional association plots for shared locus in chromosome 12.

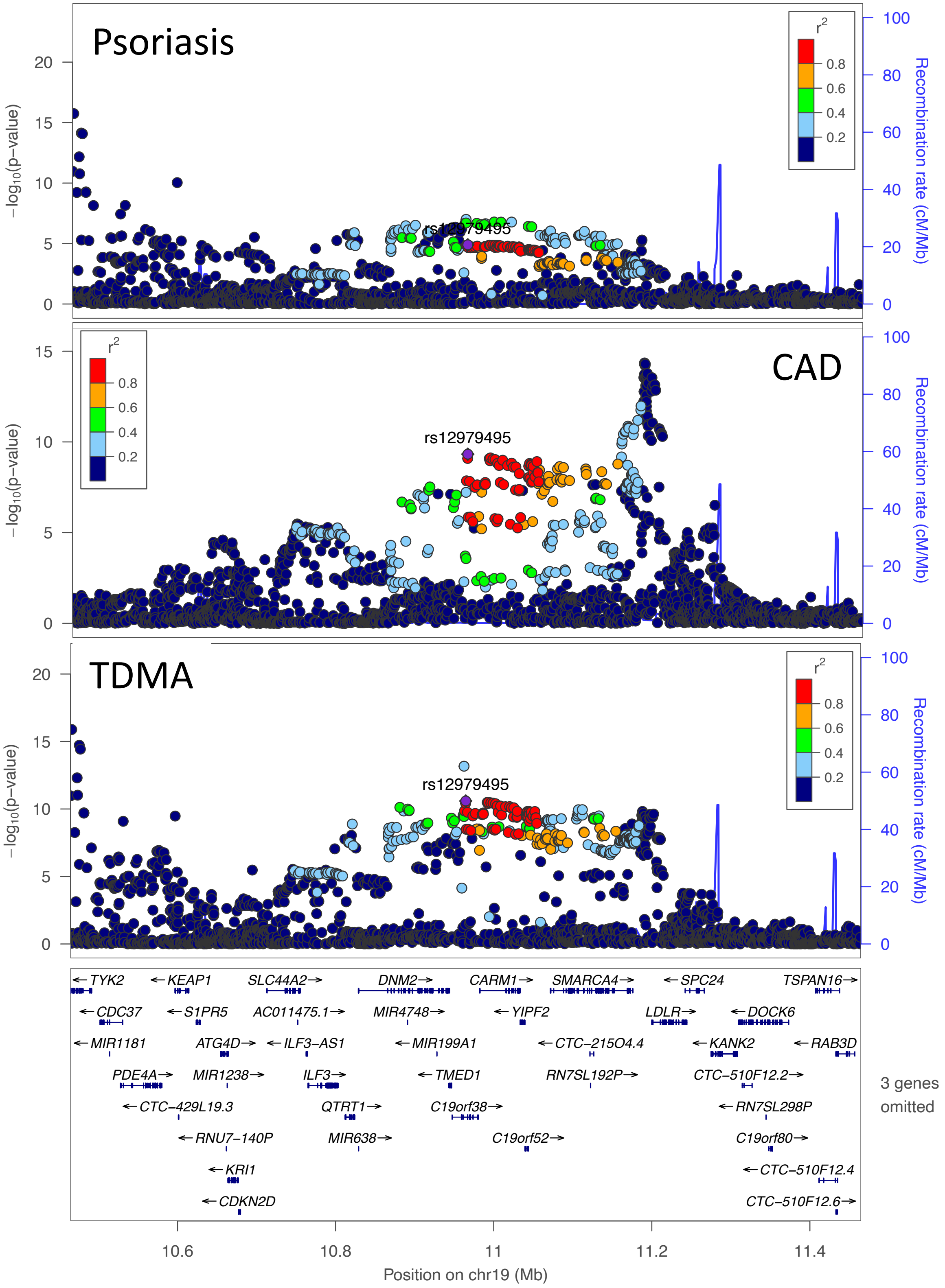

Supplementary Figure 4: Regional association plots for shared locus in chromosome 19.

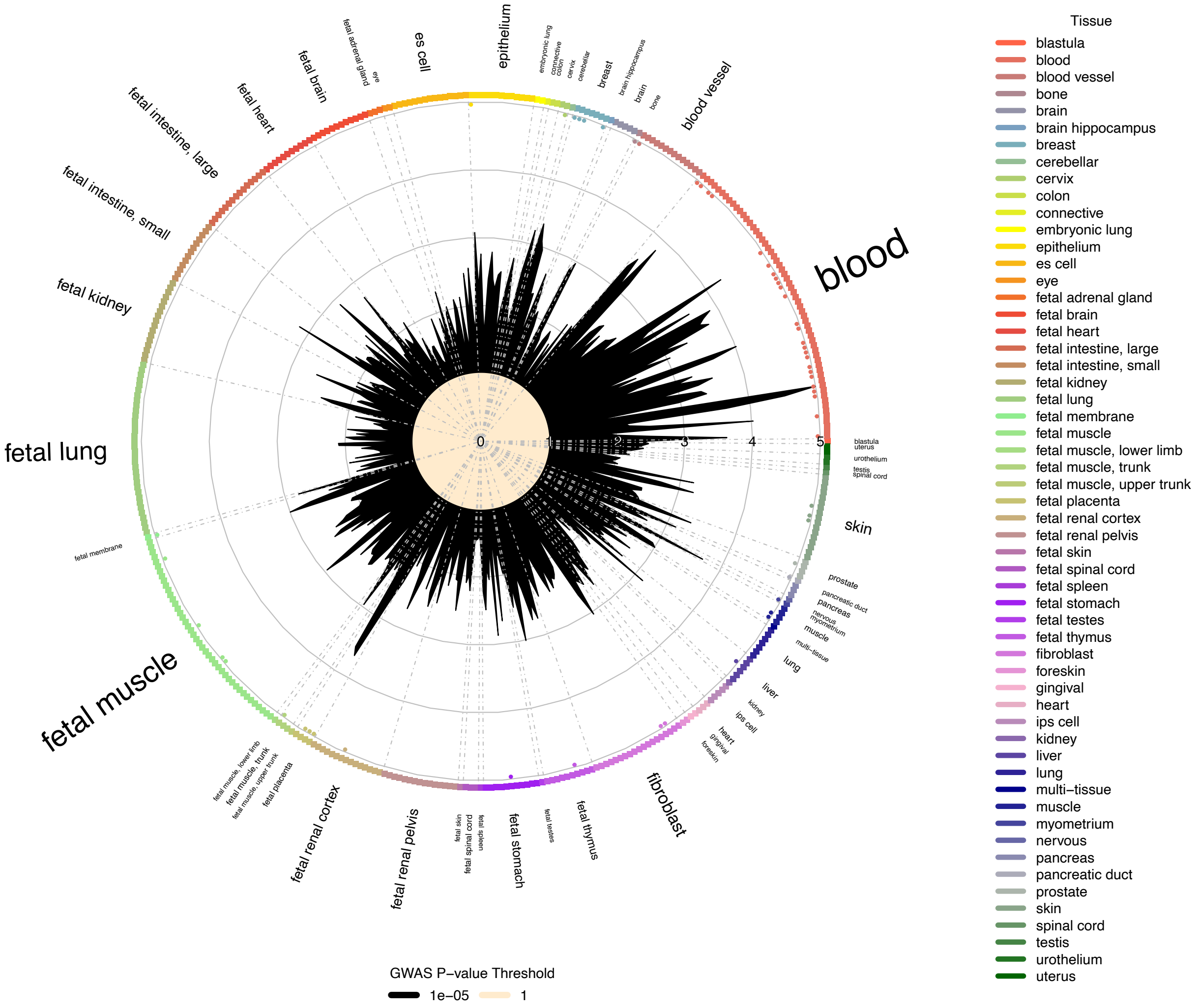

Supplementary Figure 5: Significant peaks from GARFIELD enrichment analysis.

Supplementary Table 1: Pairwise genetic correlations from LDSC.

Pre-prepared data as used from the UK Biobank for: asthma (41,934 cases, 319,207 controls); body mass index, BMI (359,983 individuals); coronary artery disease, CAD (8,239 cases, 352,902 controls), glaucoma (3,786 cases, 357,355 controls); high cholesterol, HighChol (43,957 cases, 317,184 controls); psoriasis (4,192 cases, 356,949 controls); type 2 diabetes, T2D (2,292 cases, 358,849 controls); ulcerative colitis, UC (1,916 cases, 35,9225 controls). Abbreviations are as follows: r_g, genetic correlation; p, p-value.

Trait 1	Trait 2	r_g	P
Asthma	BMI	0.174	4.9×10^{-15}
Asthma	CAD	0.135	9.3×10^{-4}
Asthma	Glaucoma	0.075	0.12
Asthma	HighChol	0.091	3.8×10^{-3}
Asthma	Psoriasis	0.049	0.56
Asthma	T2D	0.097	0.15
Asthma	UC	0.066	0.48
BMI	CAD	0.351	1.1×10^{-30}
BMI	Glaucoma	0.040	0.27
BMI	HighChol	0.319	8.9×10^{-17}
BMI	Psoriasis	0.123	0.03
BMI	T2D	0.632	2.3×10^{-14}
BMI	UC	0.010	0.84
CAD	Glaucoma	0.046	0.53
CAD	HighChol	0.602	7.4×10^{-36}
CAD	Psoriasis	0.142	0.15
CAD	T2D	0.392	1.6×10^{-4}
CAD	UC	0.154	0.12
Glaucoma	HighChol	-0.005	0.93
Glaucoma	Psoriasis	-0.153	0.16
Glaucoma	T2D	0.048	0.69
Glaucoma	UC	0.025	0.83
HighChol	Psoriasis	0.142	0.08
HighChol	T2D	0.472	9.2×10^{-7}
HighChol	UC	-0.061	0.44
Psoriasis	T2D	0.166	0.35
Psoriasis	UC	0.174	0.32
T2D	UC	0.029	0.85

Supplementary Table 2: Genes identified for psoriasis/CAD from Open Targets.
Abbreviations are as follows: CAD, coronary artery disease; n, number of genes with overall score ≥0.1.

Psoriasis/CAD (n=101)	Psoriasis-only (n=358)	CAD-only (n=740)
ACTR2, ADIPOQ,	ABHD5, ABI1, ACTA2, ADAD1, ADCY3, ADCY7,	ABCA1, ABCC8, ABCG5, ABCG8, ABHD2, ABO, ACAD11, ACE, ACHE, ACKR4, ACLY, ACTRT2,
ADORA3, ALDH2,	ADGRL2, AHR, AIM2, ANKRD30A, ANKRD55,	ACYP1, ADAMTS3, ADAMTS7, ADAMTSL4, ADCY9, ADIPOR1, ADORA1, ADORA2A, ADORA2B,
ATP4A, BACH2,	ANTXR2, AP1S3, ASAP2, ATG16L1, ATP6V1G3,	ADRA1A, ADRA1B, ADRA1D, ADRA2A, ADRA2B, ADRA2C, ADRB1, ADRB2, ADRB3, ADTRP,
BANK1, CCL2,	B3GALT6, B3GNT2, BAK1, BPTF, C10orf55,	AGER, AGT, AGTR1, AIDA, ALDH1A2, ALOX5, AMY1C, AMY2A, ANGPTL3, ANGPTL4, ANGPTL8,
CCRL2, DPP4,	C17orf67, C1orf141, C1orf68, C2orf74,	ANKDD1B, ANKRD13B, ANKRD26, ANKRD6, ANO3, AOPEP, AP4B1, APLN, APOA1, APOA5,
ESR2, FADS1,	C7orf33, CAMK2G, CAMP, CARD14, CARD9,	APOB, APOC1, APOC3, APOE, APOM, AR, ARHGAP15, ARHGAP26, ARHGAP42, ARHGEF12,
FADS2, FAP,	CASR, CCDC88B, CCHCR1, CCL11, CCL20, CCL21,	ARHGEF16, ARHGEF26, ARID4A, ARL4C, ARNTL, AS3MT, ASAH1, ASZ1, ATG16L2, ATP1B1,
FKBP1A, FOXC1,	CCL7, CCN1, CCNY, CCR5, CD2, CD226, CD28,	ATP2B1, ATP5MC1, ATP6V1B2, ATXN2, B9D2, BACH1, BAG2, BAZ1B, BCAP29, BCAS3,
GCKR, GPD2,	CD4, CD40, CD6, CD80, CD86, CD8A, CDC37,	BCL2L15, BCL3, BDNF, BET1L, BLNK, BMP1, BMPR1B, BNC2, BORCS7, BORCS7-ASMT, BRD2,
GUCY1A1,	CDK12, CDKAL1, CEP72, CFL1, CHST10, CLCN6,	BRD3, BRD4, BRDT, BROX, BSND, BTD, BTLA, BUD13, C11orf58, C1QTNF1, C1QTNF9, C1S, C2,
HMGCR, IFIH1,	CLEC16A, CLN3, CNPY2, COG6, COQ10A,	C21orf140, C3, C5, C5orf67, C8orf34, CACNA1C, CACNA1D, CACNA1F, CACNA1S, CAD,
IL17A, IL6, IL6R,	CORO1A, CPEB4, CREM, CUL2, CXCR2, DAAM1,	CALCRL, CARF, CARS2, CCDC63, CCDC71L, CCDC85C, CCDC92, CCDC97, CCM2, CCNL1, CCT6A,
LCN2, LEP, MT-	DAP, DCTD, DDC, DDX58, DEFB4A, DENND1B,	CD109, CD180, CD19, CD200, CD36, CD72, CD79A, CD79B, CDC123, CDH13, CDKN2A,
ND1, MT-ND2,	DGKD, DGKE, DHFR, DLD, DNM2, DNMT3B,	CDKN2B, CDKN2B-AS1, CELA2A, CELSR1, CELSR2, CENPW, CERT1, CETP, CFAP161, CFB,
MT-ND3, MT-	DOCK3, EDN3, EFNA1, EGFR, ELMO1, ELOA,	CFDP1, CFTR, CHGA, CHI3L1, CHRM1, CHRM3, CHRNA1, CHRNA4, CHRNB1, CHRNB2,
ND4, MT-ND4L,	EMSY, ENSG00000258790, ENSG00000267303,	CHRNB4, CHRND, CHRNE, CHRNG, CLMP, CLOCK, CNNM2, CNR1, COBLL1, COG5, COL4A1,
MT-ND5, MT-	ENSG00000281883, ENSG00000283321,	COL4A2, COL4A4, COL6A3, COPRS, CORO1C, CORO6, CRBN, CRP, CSK, CST3, CTAGE1, CUL4A,
ND6, NDUFA1,	ENSG00000283782, ENSG00000285082,	CUX2, CX3CR1, CXCL12, CXCL8, CYP1A1, CYP46A1, CYSLTR1, DAB2IP, DAGLB, DCLK2,
NDUFA10,	ERAP1, ERN1, ERP29, ETS1, EXOC2, FAM118A,	DCLRE1B, DDB1, DDI1, DDX59, DHX36, DHX38, DHX58, DOCK5, DOK7, DRC3, DRD1, DUS4L,
NDUFA11,	FAM205C, FAS, FASLG, FCGR2A, FIGNL1,	E2F1, E2F2, E2F3, E2F4, E2F5, E2F6, E2F7, E2F8, EBF1, EDN1, EDNRA, EHBP1, EHBP1L1,
NDUFA12,	FOSL2, FOXF2, FOXO1, FUBP1, FUT2, GAL3ST2,	EIF2B2, ELL, ELL3, EML1, ENSG00000243696, ENSG00000244255, ENSG00000255730,
NDUFA13,	GALC, GCA, GNA12, GPALPP1, GPR18, GPR183,	ENSG00000256966, ENSG00000262633, ENSG00000264545, ENSG00000284686, EPB41L5,
NDUFA2,	GPR35, GPR65, GRB10, GSN, HBS1L, HDAC7,	ESR1, EXOC3L2, F10, F2, F2R, F9, FAAH, FABP4, FAM177B, FAM30A, FAM3C, FARP1, FBN1,
NDUFA3,	HLA-B, HLA-C, HLA-DQA1, HLA-DRB1, HNF4A,	FBRSL1, FCHO1, FCRL1, FCRL2, FCRLA, FDX1, FER, FERD3L, FES, FGD5, FGD6, FGF21, FGF23,
NDUFA4,	HRH4, HRNR, HSD3B7, ICAM2, ICOSLG, IFI16,	FGF5, FHIT, FHL1, FHL3, FIGN, FLT1, FN1, FNDC3B, FOXB2, FOXC2, FOXL1, FRMD5, FTO,
NDUFA4L2,	IFNG, IFNGR2, IFNLR1, IFT46, IKBKE, IKZF1,	FURIN, FUT1, GABRA1, GABRA2, GABRA3, GABRA4, GABRA5, GABRA6, GABRB1, GABRB2,
NDUFA5,	IKZF3, IL10, IL12B, IL12RB2, IL13, IL17F,	GABRB3, GABRD, GABRE, GABRG1, GABRG2, GABRG3, GABRP, GABRQ, GALNT4, GCK,
NDUFA6,	IL17RA, IL18R1, IL19, IL1RL1, IL2, IL21, IL22,	GDF15, GDPD5, GEM, GGCX, GHSR, GIGYF2, GIP, GIT1, GLCCI1, GLP1R, GLRA1, GOSR2,
NDUFA7,	IL23A, IL23R, IL27, IL2RA, IL36RN, IL4R, IL6ST,	GPR149, GPR22, GRB14, GRIN1, GRIN2A, GRIN2B, GRIN2C, GRIN2D, GRIN3A, GRIN3B,
NDUFA8,	INAVA, INS-IGF2, IP6K3, IPMK, IRF1, IRF5,	GSTM1, GUCY1B1, GUCY1B2, HAPLN3, HCN4, HDAC9, HDGFL1, HECTD4, HGFAC, HHAT,
NDUFA9,	IRF8, IRGM, ITGAL, ITLN1, ITPRID1, JAK1, JAK2,	HHIPL1, HLA-DOA, HMOX1, HNF1A, HNRNPUL1, HORMAD1, HOXC4, HOXC5, HP, HPR, HRH2,
NDUFAB1,	JAK3, JAZF1, KCNH7, KEAP1, KIAA1109, KLF13,	HS3ST1, HSD17B12, HSPA1B, HSPA8, HTRA1, IBTK, ICA1L, ICAM1, IGF2R, IL16, IL18, IL1B,
NDUFAF1,	KLRC4-KLRK1, KPRP, KRT10, KSR1, LACC1,	IL33, IL37, ILRUN, INPP5B, INSIG1, INSR, IPO9, IRAG1, IRS1, IRX1, ITGA2B, ITGB3, ITGB5,
NDUFAF2,	LCE3A, LCE3B, LCE3C, LCE3D, LCE3E, LINC02694,	ITIH4, ITPK1, JCAD, KALRN, KANK2, KCNAB1, KCNE2, KCNG1, KCNH8, KCNJ11, KCNJ13,
NDUFAF3,	LINC02929, LONRF2, LRP5, LRRC32, LRRC43,	KCNK10, KCNK18, KCNK2, KCNK3, KCNK5, KCNK9, KCNN2, KCTD10, KDR, KIF6, KLF2, KLF4,
NDUFAF4,	LRRC7, LRRK2, LTBR, MAP3K8, MFSD4B,	KLF8, KLHL14, KLHL35, KMO, KSR2, L3MBTL1, LAMA5, LDLR, LIMCH1, LINC02881, LIPA, LIPC,
NDUFB1,	MMP16, MST1, MTCL1, MUC19, MUC22,	LIPG, LMOD1, LOX, LOXL1, LPA, LPIN3, LPL, LRATD2, LRP1, LRP6, LRRC10B, LYRM2, MAD1L1,
NDUFB10,	MUCL3, NAA25, NDFIP1, NFIB, NFKBIA, NFKBIZ,	MAD2L1, MAGED4, MALAT1, MAML3, MAP1B, MAP1S, MAP3K1, MAP3K14, MAP3K7CL,
NDUFB11,	NKX2-3, NOS2, NPEPPS, NPPA, NR5A2,	MAP9, MAPK11, MAPK14, MARK3, MARK4, MAT2A, MBTD1, MC4R, MCF2L, MEF2A, MERTK,
NDUFB2,	NSMCE1, NTRK1, NUFIP1, NXPE1, NXPE3,	MFGE8, MGAM, MGST2, MIA3, MICAL3, MITF, MLH3, MLXIPL, MMP2, MMP3, MMP9,
NDUFB3,	NXPE4, OLIG3, OSGIN2, OSMR, PARK7, PDGFB,	MORF4L1, MORN1, MPO, MRAS, MRPS6, MRTFA, MS4A1, MSL2, MTAP, MTHFD1L, MTHFR,
NDUFB4,	PGBD1, PHC3, PHF24, PHTF1, PI3, PKIA, PLAU,	MTHFSD, MX1, MYH11, MYL2, N4BP2L1, N4BP2L2, NAT2, NBEAL1, NCALD, NEBL, NEK9, NGF,
NDUFB5,	PLCL2, POLI, POM121L2, PPARD, PPIA, PPIF,	NME7, NME9, NOS3, NPC1L1, NPPB, NPR1, NR2C1, NR3C2, NUDT5, OAS1, OCRL, OIT3, OLR1,
NDUFB6,	PPP1R17, PPP3CA, PPP5C, PRDM1,	OPRL1, OPRM1, OSBPL10, OSM, P2RX5, P2RY12, PALLD, PARP12, PBX2, PCCB, PCNX3, PCSK6,
NDUFB7,	PRKCB, PRORP, PRSS16, PSMG1, PTGER4,	PCSK9, PDE10A, PDE1A, PDE1B, PDE1C, PDE2A, PDE3A, PDE3B, PDE5A, PDE6A, PDE6B,
NDUFB8,	PTPN2, PTPN22, PTPRC, PTPRN2, PUS10,	PDE6C, PDE6D, PDE6G, PDE6H, PDE7A, PDE7B, PDE8A, PDE8B, PDE9A, PDGFD, PDS5B,
NDUFB9,	RAD50, RARA, RARB, RARG, RASGRP1,	PECAM1, PEMT, PGR, PHACTR1, PHB, PHETA1, PHOX2B, PID1, PIK3CG, PKD2L1, PLA2G10,
NDUFC1,	RASIP1, REL, REV3L, RFX4, RIPK2, RMI2,	PLA2G2A, PLA2G5, PLA2G7, PLCG1, PLCG2, PLEKHG1, PLG, PLPP3, PMAIP1, PNPLA2, PON1,
NDUFC2, NDUFS1,	RNF114, RNF145, RNF186, RNH1, RORC	POU2AF1, PPARA, PPARG, PPM1G, PRDM16, PREX1, PRIM2, PRKAG2, PRKCE, PROCR, PSPH,
NDUFS2, NDUFS3,	RPS10-NUDT3, RPS6KB1, RSPH3, RSPO3,	PSRC1, PTGDR, PTGER1, PTGER2, PTGS1, PTGS2, PTPRK, PTPRT, PTX3, QPCTL, R3HDM2,
NDUFS4, NDUFS5,	RTEL1-TNFRSF6B, RUNX1, RUNX3, RXRA, RXRB,	RAB23, RAI1, RALGPS2, RARRES2, RASGEF1B, RASGRP3, RBP4, RBPMS2, RBX1, REN, REST,
NDUFS6, NDUFS7,	RXRG, S100A8, S1PR1, S1PR5, SAG, SATB1,	RETN, RFLNA, RGL3, RGS19, RHOF, RIC8A, RNF113B, RNF213, RNLS, ROCK2, RPGRIP1L,
NDUFS8,	SBNO2, SCRN1, SEMA6A, SGF29, SGSH, SKAP2,	RPH3A, SAMD3, SAV1, SAYSD1, SCARB1, SCML4, SCN10A, SCN11A, SCN1A, SCN2A, SCN3A,
NDUFV1,	SLC26A11, SLC26A3, SLC39A11, SLC45A1,	SCN4A, SCN5A, SCN7A, SCN8A, SCN9A, SCNN1A, SELP, SEMA5A, SEMA5B, SERPINA1,
NDUFV2,	SLC6A2, SLC6A3, SLC9A3, SLC9A8, SLIT3,	SERPINC1, SERPINE1, SERPINH1, SETD1B, SETD9, SF3A3, SFTA2, SH2D4B, SH3PXD2A, SHISA4,
NDUFV3, NFKB1,	SMURF1, SNAI1, SNAPC4, SOCS1, SOX14, SOX4,	SHOC1, SHROOM3, SIK3, SIPA1, SIRT1, SKI, SLC12A1, SLC12A3, SLC18A1, SLC18A2, SLC22A1,
NR3C1, PDE4A,	SP140, SP140L, SPIN1, SPRED2, STAT2, STAT3,	SLC22A2, SLC22A3, SLC29A1, SLC2A12, SLC35G2, SLC5A2, SLC5A3, SLCO1B1, SLN, SMAD7,
PDE4B, PDE4C,	STX1B, SULT1A2, SYNE2, TAGAP, TBC1D5,	SMARCA1, SMARCA4, SMG6, SMIM11, SMUG1, SNF8, SNHG32, SNORC, SNX22, SOD2,
PDE4D, PKIG,	TBL1XR1, THADA, THEMIS, TMEM17,	SORBS2, SORT1, SOX6, SPC24, SPECC1L, SPECC1L-ADORA2A, SPIB, SPP1, SREBF1, SRFBP1,
PLCL1, PTPN11,	TMEM171, TMEM174, TNFAIP3, TNFRSF14,	SRSF6, SSU72P8, STAG1, STAG3, STARD5, STAT6, STN1, SUMF2, SVEP1, SWAP70, TAB2,
RAB12, RDX,	TNFRSF1A, TNFRSF6B, TNFSF15, TNFSF18,	TBX20, TBXAS1, TCEA2, TCF21, TCF7L2, TDRD15, TDRD6, TDRKH, TENT5A, TFPI, TGFB1,
SH2B3, SLC22A4,	TNIP1, TNRC18, TP63, TRAF3IP2, TRIM47,	THRA, THRB, THSD7A, TLCD5, TLR7, TLR9, TM6SF2, TMEM106B, TMEM170A, TMEM200A,
SLC6A4, SMAD3,	TRPC3, TSC22D1, TTC33, TYK2, UBAC2,	TMEM91, TMPRSS2, TNFRSF13C, TNFRSF17, TNFSF4, TNNC1, TNNI3, TNNT2, TNS1, TNXB,
TLR4, TNF, TRIB1,	UBASH3A, UBE2E3, UBE2L3, UBLCP1,	TOMM40, TOMM5, TOP1, TRAF5, TRIM5, TRPA1, TSBP1, TSPAN13, TSPAN2, TTC29, TTC32,
TRPV1, TSPAN14,	UGT1A8, USP12, USP25, USP34, YDJC	TUBA3C, TUBB, TUBB1, TUBB2A, TUBB2B, TUBB3, TUBB4A, TUBB4B, TUBB6, TUBB8, TWIST1,
VDR, VEGFA,	ZBTB40, ZC2HC1A, ZC3H12C, ZFP90, ZFP91,	TXNIP, TXNL4B, UBE2Z, UBE3B, UMPS, UNC5C, USP24, VAMP5, VAMP8, VKORC1, VPREB3,
ZEB2, ZNF365,	ZFYVE16, ZKSCAN8, ZMIZ1, ZNF366	VPS11, VWDE, VWF, WDR11, WDR12, WIPI1, WT1, WWP2, XDH, YBX3, ZC2HC1C, ZC3HC1,
ZNF831	ZNF804A, ZPBP2, ZSCAN23	ZFHX3, ZFPM2, ZHX3, ZNF32, ZNF335, ZNF507, ZNF652, ZNF711, ZNF827, ZPR1, ZRANB3

Supplementary Table 3: Proportion of Open Target genes involved in systemic inflammation.
Genes are selected that have overall score ≥0.1. Abbreviations are as follows: CAD, coronary artery disease.

	**Psoriasis/CAD**	**Psoriasis-only**	**CAD-only**
Immune	45 (45%)	92 (26%)	114 (15%)
Non-immune	56 (55%)	266 (74%)	626 (85%)
Total	101 (100%)	358 (100%)	740(100%)

Supplementary Table 4: Replication and PheWAS for TDMA loci in UK Biobank.

PheWAS conducted using SAIGE on TopMed imputed genetic data. The numbers indicated in brackets are PheCodes. In cases where nested PheCodes are significant, we keep the more specific subcodes.

**Locus**	**Coronary atherosclerosis (411.4)**		**Psoriasis vulgaris (696.41)**		**Other top three most significant PheWAS codes (excluding 411.4 and 696.41)**
	OR	*P*	*OR*	*P*	
rs6430076-G	1.06	$1.1x10^{-6}$	0.90	$2.7x10^{-3}$	Myocardial infarction (411.2): OR=1.06, p=$6.0x10^{-5}$; Cancer of connective tissue (170.2): OR=1.35, p=$1.9x10^{-4}$; Poisoning by hormones and synthetic substitutes (962): OR=1.27, p=$1.6x10^{-3}$
rs1990760-T	1.03	$2.2x10^{-3}$	1.11	$3.3x10^{-3}$	Hypothyroidism NOS (244.4): OR=1.08, p=$8.5x10^{-10}$; Unstable angina (411.1): OR=1.07, p=$7.0x10^{-4}$; Psoriatic arthropathy (696.42): OR=1.20, p=$1.4x10^{-3}$
rs76151170-C	1.03	0.21	1.27	$7.0x10^{-5}$	Fracture of unspecified bones (809): OR=0.76, p=$3.4x10^{-4}$; Tympanosclerosis and middle ear disease related to otitis media (385.5): OR=0.51, p=$3.8x10^{-4}$; Fracture of vertebral column without mention of spinal cord injury (805): OR=0.79, p=$2.5x10^{-3}$
rs12979495-G	1.05	$3.7x10^{-5}$	1.04	0.31	Hypercholesterolemia (272.11): 1.04, p=$1.4x10^{-5}$; Colorectal cancer (153): OR=1.09, p=$3.4x10^{-4}$; Pituitary hypofunction (253.2): 1.38, p=$1.4x10^{-3}$
