# Supplementary material for: Shared genetic risk factors and causal association between psoriasis and coronary artery disease
Source: Nat Commun. 2022 Nov 2;13:6565. doi: 10.1038/s41467-022-34323-4 (PMC9630428; doi:10.1038/s41467-022-34323-4)
Supplement: Supplementary file 2 — Reporting Summary [file 41467_2022_34323_MOESM2_ESM.pdf]

nature portfolio

Corresponding author(s): Lam C. Tsoi

Last updated by author(s): Sep 27, 2022

Reporting Summary

Nature Portfolio wishes to improve the reproducibility of the work that we publish. This form provides structure for consistency and transparency in reporting. For further information on Nature Portfolio policies, see our Editorial Policies and the Editorial Policy Checklist.

Statistics

For all statistical analyses, confirm that the following items are present in the figure legend, table legend, main text, or Methods section.

n/a	Confirmed	
☐	☒	The exact sample size (*n*) for each experimental group/condition, given as a discrete number and unit of measurement
☒	☐	A statement on whether measurements were taken from distinct samples or whether the same sample was measured repeatedly
☐	☒	The statistical test(s) used AND whether they are one- or two-sided<br>*Only common tests should be described solely by name; describe more complex techniques in the Methods section.*
☐	☒	A description of all covariates tested
☐	☒	A description of any assumptions or corrections, such as tests of normality and adjustment for multiple comparisons
☐	☒	A full description of the statistical parameters including central tendency (e.g. means) or other basic estimates (e.g. regression coefficient) AND variation (e.g. standard deviation) or associated estimates of uncertainty (e.g. confidence intervals)
☐	☒	For null hypothesis testing, the test statistic (e.g. *F*, *t*, *r*) with confidence intervals, effect sizes, degrees of freedom and *P* value noted<br>*Give P values as exact values whenever suitable.*
☒	☐	For Bayesian analysis, information on the choice of priors and Markov chain Monte Carlo settings
☒	☐	For hierarchical and complex designs, identification of the appropriate level for tests and full reporting of outcomes
☐	☒	Estimates of effect sizes (e.g. Cohen's *d*, Pearson's *r*), indicating how they were calculated

Our web collection on statistics for biologists contains articles on many of the points above.

Software and code

Policy information about availability of computer code

Data collection	No software was used
Data analysis	LDSC v1.01; COLOC v5.1.0; GCTA v1.92.1; GARFIELD v2; TwoSampleMR v0.4.26; GRAPPLE v0.2.0; PLINK v1.9

For manuscripts utilizing custom algorithms or software that are central to the research but not yet described in published literature, software must be made available to editors and reviewers. We strongly encourage code deposition in a community repository (e.g. GitHub). See the Nature Portfolio guidelines for submitting code & software for further information.

Data

Policy information about availability of data

All manuscripts must include a data availability statement. This statement should provide the following information, where applicable:

- Accession codes, unique identifiers, or web links for publicly available datasets
- A description of any restrictions on data availability
- For clinical datasets or third party data, please ensure that the statement adheres to our policy

Summary statistics for the coronary artery disease GWAS are available online (http://www.cardiogramplusc4d.org), along with the BMI and waist-hip ratio GWAS (https://portals.broadinstitute.org/collaboration/giant/index.php/GIANT_consortium), and the LDSC-formatted UK Biobank summary statistics (http://www.nealelab.is/uk-biobank). HDL cholesterol (GCST007140 [https://www.ebi.ac.uk/gwas/studies/GCST007140]), LDL cholesterol (GCST007141 [https://www.ebi.ac.uk/gwas/studies/GCST007143]), total cholesterol (GCST007143 [https://www.ebi.ac.uk/gwas/studies/GCST007143]) and triglyceride (GCST007142

[https://www.ebi.ac.uk/gwas/studies/GCST007142]) GWAS summary statistics are available from the European Bioinformatics Institute. Data for three of the psoriasis cohorts (phs000019.v1.p1 [https://www.ncbi.nlm.nih.gov/projects/gap/cgi-bin/study.cgi?study_id=phs000019.v1.p1]; phs001306.v1.p1 [https://www.ncbi.nlm.nih.gov/projects/gap/cgi-bin/study.cgi?study_id=phs001306.v1.p1]; phs000982.v1.p1 [https://www.ncbi.nlm.nih.gov/projects/gap/cgi-bin/study.cgi?study_id=phs000982.v1.p1]) are available in dbGap, with data for the other cohorts available upon reasonable request. Access to data from the Michigan Genomics Initiative can be requested through the JIRA ticketing system (https://precisionhealth.umich.edu/our-research/michigangenomics/). eQTL data from eQTLGen (https://www.eqtlgen.org), CAGE (https://shiny.cnsgenomics.com/CAGE/), NESDA/NTR (https://eqtl.onderzoek.io/), Westra et al. (https://genenetwork.nl/bloodeqtlbrowser/) and GTeX (https://gtexportal.org/), as well as the mQTL datasets Hannon et al. and McRae et al. (https://cnsgenomics.com/software/smr/#DataResource) and mQTLdb (http://www.mqtldb.org) are available online. Expression data for psoriasis (GSE121212 [https://www.ncbi.nlm.nih.gov/geo/query/acc.cgi?acc=GSE121212]) and CAD (GSE23561 [https://www.ncbi.nlm.nih.gov/geo/query/acc.cgi?acc=GSE23561]) are available through GEO. The Mouse Genome Informatics database is available online (http://www.informatics.jax.org), as is the 3D-genome Interaction Viewer (http://kobic.kr/3div).

Human research participants

Policy information about studies involving human research participants and Sex and Gender in Research.

Reporting on sex and gender	Gender is used only as a covariate for the epidemiological analysis
Population characteristics	Race, age, gender, body mass index (BMI) and socioeconomic disadvantage were used as covariates for the epidemiological analysis. Diagnosis was determined using ICD-9/10 codes (ICD-9 691.0 or 696.1, or any of the L40 ICD-10 sub-codes for psoriasis; any of the 414 ICD-9 sub-codes, or I25 ICD-10 sub-codes for CAD)
Recruitment	No recruitment occurred, as all analysis was on secondary data
Ethics oversight	The Michigan Genomics Initiative study was approved by the University of Michigan institutional review board

Note that full information on the approval of the study protocol must also be provided in the manuscript.

Field-specific reporting

Please select the one below that is the best fit for your research. If you are not sure, read the appropriate sections before making your selection.

☒ Life sciences ☐ Behavioural & social sciences ☐ Ecological, evolutionary & environmental sciences

For a reference copy of the document with all sections, see nature.com/documents/nr-reporting-summary-flat.pdf

Life sciences study design

All studies must disclose on these points even when the disclosure is negative.

Sample size	11,024 psoriasis and 60,801 CAD patients, along with accompanying controls for trans-disease meta-analysis. Since we used summary statistics from previous studies, we did not estimate in advance what the sample size should be, but used what was available.
Data exclusions	No data were excluded from the analysis
Replication	We replicated our findings using summary statistics from an additional CAD GWAS. This replication analysis was performed once, using the dataset that was available
Randomization	Not relevant to our study as no allocation occurred (we only analyzed secondary data)
Blinding	Not relevant to our study as we only analyzed secondary data

Reporting for specific materials, systems and methods

We require information from authors about some types of materials, experimental systems and methods used in many studies. Here, indicate whether each material, system or method listed is relevant to your study. If you are not sure if a list item applies to your research, read the appropriate section before selecting a response.

Materials & experimental systems

n/a	Involved in the study
☒	☐ Antibodies
☒	☐ Eukaryotic cell lines
☒	☐ Palaeontology and archaeology
☒	☐ Animals and other organisms
☒	☐ Clinical data
☒	☐ Dual use research of concern

Methods

n/a	Involved in the study
☒	☐ ChIP-seq
☒	☐ Flow cytometry
☒	☐ MRI-based neuroimaging
